# A Laser Photoacoustic Analysis of Residual CO_2_ and H_2_O in Larch Stems

**DOI:** 10.3390/bios5010001

**Published:** 2014-12-23

**Authors:** Boris Ageev, Yurii Ponomarev, Valeria Sapozhnikova, Dmitry Savchuk

**Affiliations:** 1 V. E. Zuev Institute of Atmospheric Optics, Siberian Branch of the Russian Academy of Sciences, 1 Academician Zuev Square, Tomsk 634021, Russia; E-Mails: ageev@asd.iao.ru (B.A.); yupon@iao.ru (Y.P.); 2 Institute of Monitoring of Climatic and Ecological Systems, Siberian Branch of the Russian Academy of Sciences, 10/3 Academichesky Prospekt, Tomsk 634055, Russia; E-Mail: savchuk@imces.ru

**Keywords:** annual tree rings, larch, CO_2_, H_2_O, climate response

## Abstract

Every so often, the results obtained from investigations into the effects of varying environmental conditions on the tree growth rate at the same sites and on the change in the carbon balance in plants, using traditional methods, are found to differ widely. We believe that the reason for the ambiguity of the data has to do with failure to account for the role of the residual CO_2_ (and H_2_O) in the tree wood exhibiting a climate response. In our earlier work, the results of a laser photoacoustic gas analysis of CO_2_ and H_2_O vacuum-desorbed from disc tree rings of evergreen conifer trees were presented. In this paper, laser photoacoustic measurements of tree ring gases in deciduous conifer trees and CO_2_ carbon isotope composition determined by means of a mass spectrometer are given. Conclusions are made regarding the response of annual larch CO_2_ disc tree ring distributions to climatic parameters (temperatures and precipitation). The data about the CO_2_ disc content for different sites are compared.

## 1. Introduction

Environmental changes (temperature, atmospheric CO_2_ rise and variations in the CO_2_ carbon isotope composition) are one of the currently central problems now [1]. An assessment of these changes with reasonable accuracy is only possible if there is information about the past climate. This kind of information can be acquired by climate reconstruction from the tree ring width and density as functions of the environment temperature. The temperature variations correlate well with the tree growth characteristics measured in 1880–1960. However, since 1960, the correlation for high latitudes has been broken: while the environment temperature continued to rise, the annual tree ring width decreased, and the divergence problem, as it is called, arose [2]. Another problem was associated with the effect of the atmospheric CO_2_ rise (incidentally, the latter was the same everywhere) on plants. It was expected that plants would exhibit the same response to CO_2_ rise, and this would happen in plants growing in the same conditions, to say the least. That was not the case, however [3]. Extensive literature has been devoted to the influence of excess CO_2_ on the tree growth (see e.g., [4]), but “there is no empirical evidence for a long-term, sustained stimulation of the tree growth by elevated CO_2_ in natural undisturbed setting with a natural steady state nutrient cycle” [5] (p. 1096).

The discordance between the data may be due to neglect of the CO_2_ wood tree ring storage. We believe that findings of our studies on annual CO_2_ (H_2_O) variations in disc tree rings will make it possible to look at the foregoing problems from other points of view. It is known that the stem cell respired CO_2_ plays an important role in the tree carbon balance. It is also common knowledge that most of CO_2_ in stems originates from respiring cells in stems and roots and exhibits diurnal and seasonal variability [6]. However, the behavior of the stem CO_2_ and H_2_O distributions, the annual tree ring CO_2_ content and, especially, the response of the СО_2_ distributions to climate change have been totally unexplored. We have performed an extensive analysis of wood tree ring gas samples of evergreen conifers (1700 gas samples of discs from different sites) and found that (1) considerable portions of CO_2_ and transpirational H_2_O are stored in annual ring wood; (2) the CO_2_ carbon isotope composition in the samples studied differs from the atmospheric CO_2_ carbon isotope composition; (3) annual CO_2_ rise in disc tree rings is observed; (4) the CO_2_ (and H_2_O) chronologies are characterized by pronounced cyclicity. We have studied the CO_2_ (and H_2_O + CO_2_) variations in Siberian stone pine and spruce disc tree rings with the tree age and found the relationship between CO_2_ and H_2_O parameters and climatic features [7,8]. The work was continued, and a laser photoacoustic gas analysis of CO_2_ and H_2_O vacuum-desorbed from disc tree rings of a deciduous conifer tree (larch) has been performed. The СО_2_ annual distributions in the disc tree rings of a 300 year old Siberian larch (*Larix sibirica* Ledeb.) from Tomsk Oblast, Russia and a larch from Lake Baikal have been studied. The results of CO_2_ carbon isotope composition measurements and analyses of the relationship between CO_2_ variations and climatic parameters have been examined.

The measurement data may be of interest to those who seek to understand how stem respiration varies with environmental conditions, to dendrochronologists and experts in dendroecology and carbon dioxide balance. Online *in situ* monitoring of CO_2_ and H_2_O cores may offer an opportunity to study forest system adaptation for climate change and provide original data about forest health in ecological risk areas. Special attention is given to the fact that CO_2_ conservation in stems has been poorly studied, and this kind of information may account for the diversity of the results obtained by different researchers and explain a number of phenomena involved. There are many papers aimed at estimating the CO_2_ release to the atmosphere by tree stems. However, to our knowledge, none of the publications deals with annual CO_2_ estimates inside stems. The objectives of this work were to show the feasibility of analysis of vacuum-desorbed residual CO_2_ and H_2_O by methods of laser photoacoustic spectroscopy and to attract the attention of the scientific community to this interesting and totally unexplored field of research.

## 2. Experimental Section

Measurements were performed of the CO_2_ (and H_2_O) content in gas samples vacuum-desorbed from the disc tree rings of deciduous conifer trees (larches) growing in different regions of Russia. Annual CO_2_ (and H_2_O) distributions were studied in discs of a Siberian larch (*Larix sibirica* Ledeb.) growing at 56°26'N and 85°03'E in Tomsk Oblast (West Siberia, Russia) and a larch disc from Lake Baikal (East Siberia, Russia). The latter was taken near the settlement of Chernorud (53°00'N, 105°24.5'E, Irkutsk Oblast, the northwest coast of Middle Baikal). Priol’khonie, where Chernorud is located, is the driest area near Lake Baikal. The radiation index of dryness corresponds to dry steppes. In certain years, early in the vegetation period (May–June), precipitation is entirely absent there, which affects the tree growth rate. Prior to the analysis, all tree disсs were stored at room temperature and humidity for a period of time ranging from 6 months to several years, thus the wood material can be considered room-dried. It should be noted that the performance of the method suggested here was tested using not only dry discs but a disc of a living tree as well.

**Figure 1:**
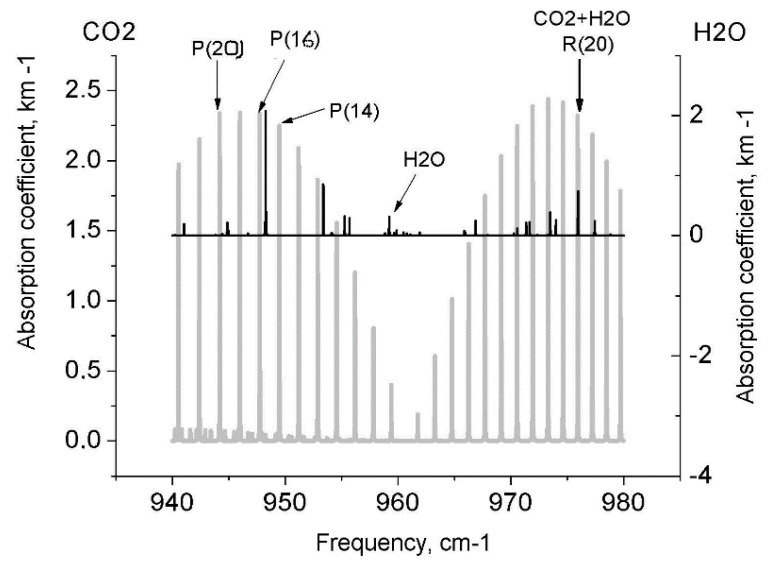
Coincidence of the CO_2_ and H_2_O absorption lines in the 10.6 µm region and the СO_2_ and H_2_O frequency dependence of the calculated absorption coefficients (km^−1^).

A laser photoacoustic analysis based on different laser sources is a modern gas detection tool used to advantage in many applications for a long time. Our experimental system and a procedure for investigating the CO_2_ and H_2_O content in gas samples extracted under vacuum from disc tree rings were described elsewhere (see, for example, [7,8]). The measurements were performed by a laser photoacoustic (PA) spectrometer using a computerized model of a tunable waveguide 10.6 µm CO_2_ laser operating in four CO_2_ laser lines: 10 *P* (20, 16, 14) coinciding with the CO_2_ absorption lines and 10 *R* (20) coinciding with the CO_2_ and water-vapor absorption lines (CO_2_ + H_2_O). The wood in each of the rings was planed down with special chisels. Samples of the same weight were placed in four exposure chambers evacuated to obtain a short-term vacuum and provide wood-sorbed gas release. In all the measurements, the gas samples (*P* = 6–8 Torr) from the exposure chambers were admitted to an evacuated PA cell. A PA signal was generated due to nonradiative de-excitation of the energy absorbed in optical transitions by the gas under study. The absorbed power was determined directly from heat, and the PA signal was generated in the gas sample. The acoustic waves were then measured by a PA cell microphone. Therefore, the PA spectrum could be correlated with the optical absorption spectra of the sample. Once the system was calibrated, *i.e.*, the absorption in gases with known concentration was measured, the calibration coefficient was found, and the absorbing component concentrations in the gas sample studied were determined. The experimental conditions (air was added to the PA cell to provide a pressure of 100 Torr) enabled us to obtain approximately the same absorption coefficients of the samples in the 10 *P* (20, 16 and 14) laser lines and provide maximum photoacoustic absorption detection sensitivity. The amplitudes ***U*** and ***U*_air_** of the signals from the mixture under investigation and air were recorded, ***ΔU*** = ***U*** − ***U*_air_** was determined, and the relative CO_2_ content in the gas sample for each disc tree ring was found, using a calibration curve. The amplitude of the photoacoustic signal was proportional to the CO_2_ and H_2_O concentrations of the absorbing components of the gas samples under study to within a constant calibration factor. The results were the mean of the measured values for the three laser lines, and the relevant correlation coefficients were 0.85–0.9. The ultimate absorption coefficient detection sensitivity of the spectrometer used was 2 × 10^−5^ cm^−1^ for a laser power of 70 mW, and the calibration measurement error was no more than ±5%. Figure 1 illustrates the coincidence of the CO_2_ and H_2_O spectra.

The results of the investigations into the annual CO_2_ (and H_2_O) variations in the larch disc tree rings were smoothed out by an 11-year running average. Fourier analysis with the use of the ORIGIN software was employed for testing periodic signals.

The analysis of the carbon isotope composition (δ^13^ С) of the desorbed CO_2_ in several annual disc tree rings was performed by a mass spectrometer. Dendroisotopic analyses are known to be widely used for detecting past changes in the air quality, evaluating the forest responses to air pollution, and accounting for the variations in the local amounts of precipitation or soil water [9]. However, we are not aware of any work dealing with the CO_2_ carbon isotope composition vacuum-desorbed from disc tree rings. It is known that leaves and tree wood are characterized by a lower carbon isotope composition (from −20‰ to −30‰), and at present, the carbon isotope composition of CO_2_ in air is about δ^13^ С = −8‰ [10].

To verify the fact that CO_2_ in the samples studied is generated by the trees themselves instead of being supplied from the atmosphere, a method was developed of an isotope analysis of carbon of the desorbed CO_2_ in N_2_ flow at *T* = 80° (precipitated as BaCO_3_). The tree ring carbon isotope composition of CO_2_ chemically extracted from the tree ring wood was determined by means of a DELTA V Advantage mass spectrometer to within ±0.5‰ for a confidence probability of 0.95. The carbon isotope composition was expressed using the (δ^13^ С = Delta 13C) notation as deviations from the internationally accepted standards, Vienna Pee Dee Belemnite (VPDB):

δ13C, (‰) = [(*R*_sample_/*R*_standard_) − 1] × 1000 (1)

where *R*_sample_ and *R*_standard_ are the ^13^C/^12^C ratios in the sample and in the standard, respectively.

## 3. Results and Discussion

### 3.1. Measurements of the Carbon Isotope Composition of the Larch Tree Ring CO_2_

**Figure 2:**
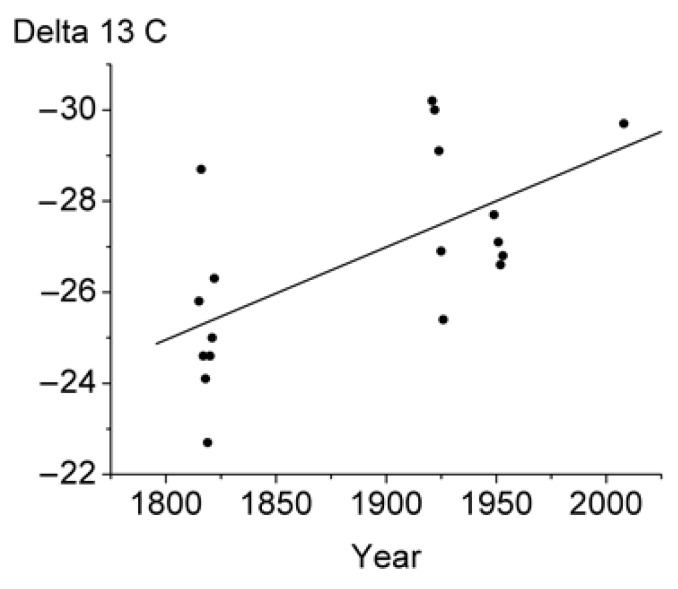
Measured annual variations in the carbon isotope composition of the vacuum-desorbed CO_2_ (Delta 13C) from the tree ring gas samples of the 300 year old larch. A fractionation effect is seen to affect the annual distribution of the vacuum-desorbed СО_2_.

By now the carbon isotope composition of 85 samples of different conifer disc tree rings has been investigated. The results obtained show that the samples are enriched in light isotope ^12^C up to δ^13^ С = −25.3‰ for spruce, varying between −25‰ (1894) and −36.4‰ (1986) for the Siberian stone pine (Tomsk Oblast, Russia) and between −26.1‰ (1886) and −27.7‰ (1995) for the spruce disc tree rings from the Altai Mountains, Russia. The tree ring carbon isotope composition of CO_2_ chemically extracted from the tree ring wood of the 300 year old larch is shown in Figure 2. The samples are enriched in light isotope ^12^C from δ^13^ С ≈ −25 ‰ to δ^13^ С ≈ −30‰. The measurements were made on the rings of 1815–1822, 1922–1926, 1949–1954, and of 2007 + 2008 (the sum of two very narrow tree rings). It is obvious that CO_2_ is formed by trees themselves due to metabolic processes at work in the trees rather than being of atmospheric origin.

**Figure 3:**
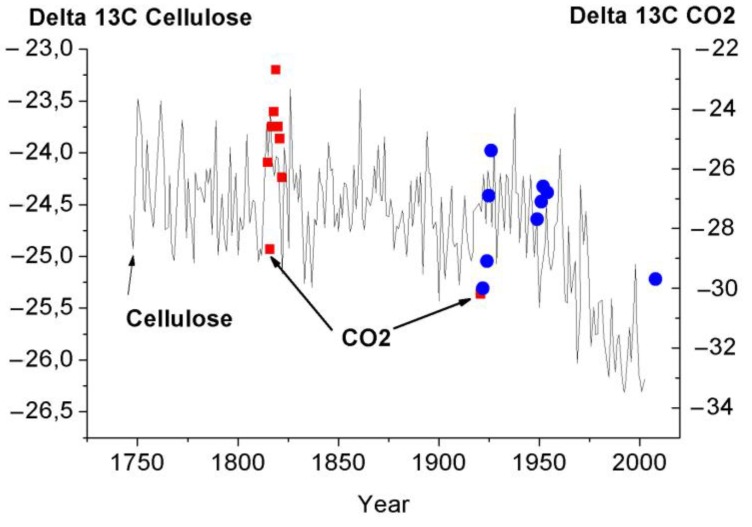
Comparison of variations in the carbon isotope composition of CO_2_ desorbed from the tree ring wood of the 300 year old larch with variation in Delta 13C of cellulose (Figure 4 from [11]. Figure 4 [11] was digitized before use).

**Figure 4:**
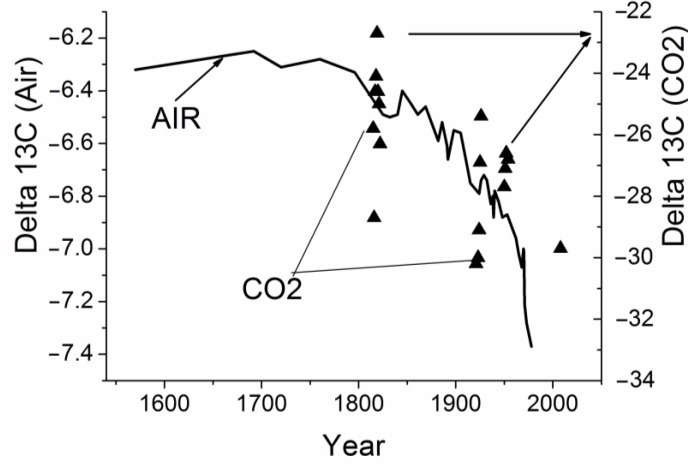
Comparison of variations in the carbon isotope composition of CO_2_ desorbed from the tree ring wood of the 300 year old larch with variations in the Delta 13C ratio in air extracted from Antarctic ice core and firn samples [12].

We thought it would of interest to compare the results obtained for the isotope composition in the cell-respired CO_2_ with variations in the cellulose carbon isotope composition [11] and with those for the atmospheric air CO_2_ [12], Figure 3 and Figure 4. Although the comparison was performed at different scales and for different objects, the results show the same tendency towards annual enrichment in light ^12^C isotope both of atmospheric air and of the carbon isotope composition of cellulose and desorbed CO_2_. We wanted to use the illustrations to emphasize the very fact that the same tendency relating to variations in Delta13C was retained in totally different objects.

**Figure 5:**
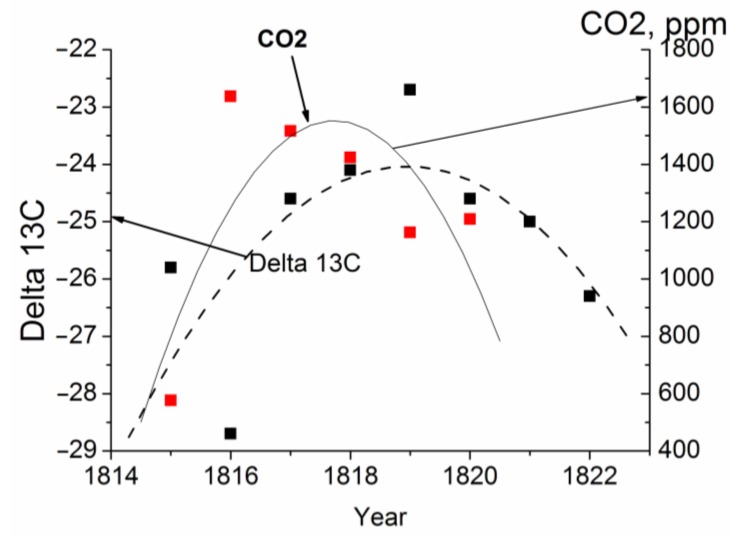
Comparison of annual distributions of the concentration of CO_2_ vacuum-desorbed from the tree ring wood (ppm) and carbon isotope composition of CO_2_ (Delta 13C) in the 300 year old larch disc for 1815–1822. The concentration of CO_2_ vacuum-desorbed from the tree ring wood in 1815–1822 is in excess of the CO_2_ concentration in air observed in recent years (~400 ppm).

There is no way to perform measurements of the CO_2_ and carbon isotope composition at the same points of the disc tree rings because of high wood material consumption. We used the neighboring tree ring sections for an analysis of these parameters. Our numerous measurements of the carbon isotope composition of the vacuum-desorbed CO_2_ have shown that an increase in the CO_2_ concentration in a gas sample brings about changes in the carbon isotope composition: the higher is the CO_2_ concentration in the sample, the lighter is the isotope composition, and vice versa. As the CO_2_ and carbon isotope composition has to be measured at different points in the tree rings, on frequent occasions, the shape of the curves is different. Variations in the annual carbon isotope composition of CO_2_ and in the vacuum-desorbed CO_2_ in the larch tree rings are illustrated in Figure 5.

### 3.2. CO_2_ and H_2_O Variations in the Larch Disc Tree Rings

**Figure 6:**
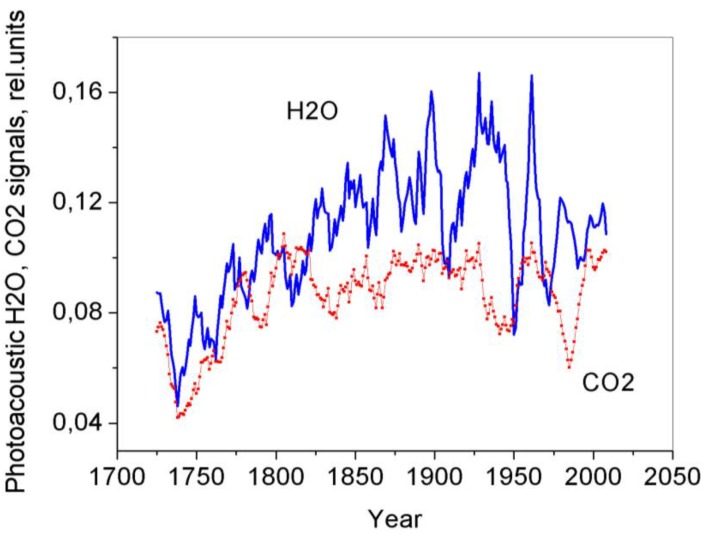
Comparison of photoacoustic signals from CO_2_ and H_2_O vacuum-desorbed from the tree rings of the 300 year old larch in the CO_2_ laser lines *P* (20) (CO_2_) and *R* (20) (H_2_O). The results were smoothed out by a 11-year running average.

It is generally believed that CO_2_ released by respiring cells in tree stems completely diffuses to the atmosphere in the course of time. However, our numerous tests of the vacuum-desorbed gas samples taken from the tree ring wood show that part of CO_2_ conserved in stems is typically dissolved in water. At the same time, H_2_O and CO_2_ are shown to be irregularly distributed in the disc tree rings. Our earlier results obtained from a laser photoacoustic gas analysis of the tree ring vacuum-desorbed CO_2_ and H_2_O from evergreen conifer trees were presented in [7,8]. All conifer discs were shown to exhibit peculiar annual CO_2_ and H_2_O tree ring distributions with distinct 4-year cyclicity. Since larches are deciduous conifer trees, there was a need to examine the peculiar behavior of the CO_2_ and H_2_O chronologies in the larch tree rings, especially for the trees cut in different regions. The results of investigations into the vacuum-desorbed CO_2_ (and H_2_O) from the tree ring wood of the 300 year old larch (Siberia) are presented in Figure 6. We could fix the absorption as the sum of two components (CO_2_ + H_2_O) in the *R* (20) CO_2_ laser line, because their absorption lines coincide (Figure 1). To obtain the H_2_O absorption alone, we subtracted the CO_2_ signals from the *R* (20) signals. As the result, two trends were observed, which enabled us to examine and compare the annual CO_2_ and H_2_O distributions in the disc tree rings of the 300 year old larch. The results were smoothed out by the 11-year running average. It follows from Figure 5 and Figure 6 that the data obtained by the method proposed here show that considerable portions of CO_2_ and H_2_O are stored in the tree stem rings and exhibit specific annual distributions. It is apparent from the figures that the CO_2_ and H_2_O distributions in the tree rings demonstrate characteristic features: (1) ~25-year H_2_O cycles; (2) ~25-year CO_2_ cycles up to ~1830; and (3) variations in the CO_2_ distribution since 1830, which may be evidence for an impact of industrial operations as atmospheric pollution sources on stem respiration.

**Figure 7:**
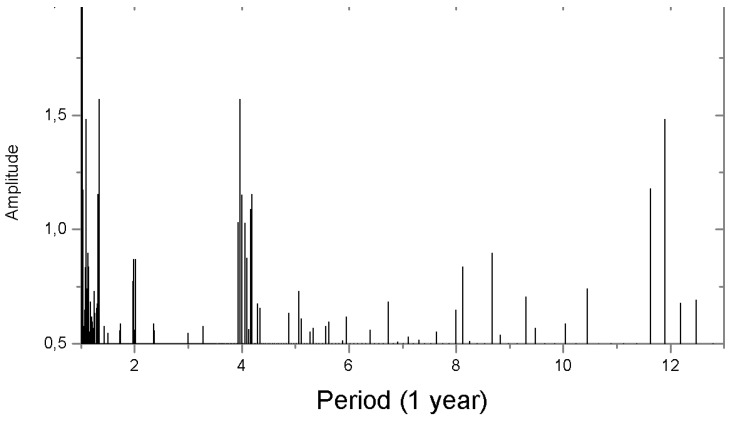
Amplitude spectrum of the tree ring CO_2_ in the 300 year old larch.

**Figure 8:**
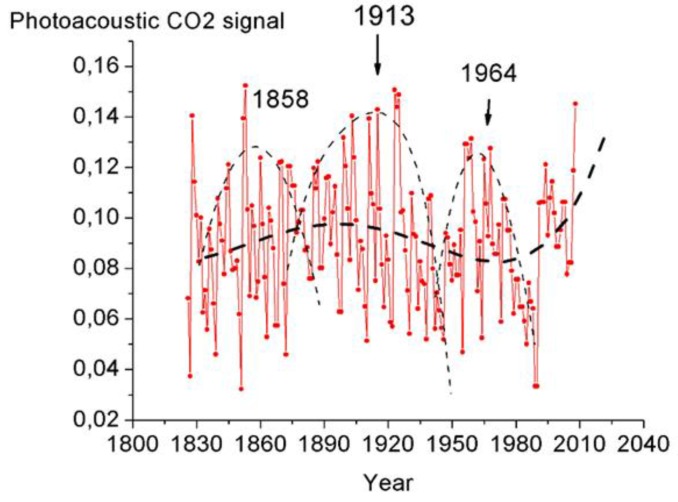
Superposition of long-term cyclicity on short-term cycles of the annual CO_2_ distribution in the disc tree rings of the 300 year old larch.

We have analyzed the measurement results for CO_2_ vacuum-desorbed from the tree rings since 1830 to find out whether the measurements showed the existence of cyclic CO_2_ variations. The results of Fourier analysis of the tree ring CO_2_ variations (1840–1935) with the use of the ORIGIN software are shown in Figure 7. Similar to the results obtained from investigations into the tree ring CO_2_ distribution in the Siberian stone pine [7,8], the tree ring CO_2_ stored in the larch disc exhibits 2-, 4-year, and higher-order cycles. To provide a more graphic illustration of the foregoing effect, we have used polynomials to approximate the tree ring CO_2_ distribution data collected in 1840–1935. The polynomial approximation of the CO_2_ tree ring distributions obtained since 1830 has emphasized ~50-year long-term cycles (Figure 8) and a tendency towards disc tree ring CO_2_ rise for the last years attributable to atmospheric CO_2_ rise [13]. These cycles (~50 year) are difficult to distinguish by the ORIGIN software.

**Figure 9:**
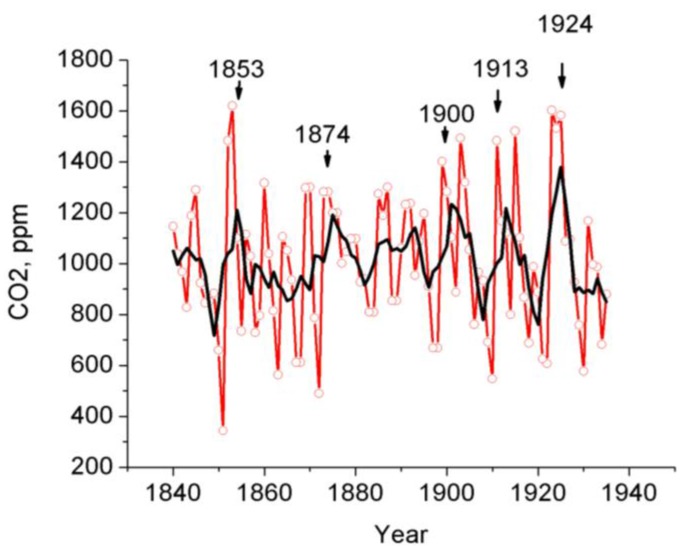
Variations in the annual vacuum-desorbed СО_2_ concentrations (ppm) in1840–1935. Appreciable variations in the CO_2_ cycles in the 300 year old larch rings are observed.

We have discovered that the cyclic patterns studied may vary. As an example, the manner in which the cyclic patterns of the CO_2_ tree ring distribution averaged over 4-year running average values varied in 1840–1935 is demonstrated in Figure 9.

Previously it was found [7,8] that the CO_2_ and H_2_O content in the tree rings of the Siberian stone pine varying over a wide range of time scales was affected by climatic factors. This is most closely related to 4-year and 2-year periods of variations in the amount of precipitation in the dormant phase, whereas long-term variations in the CO_2_ content anticorrelate with temperature in the vegetation period. As regards the 300 year old larch, a significant correlation was found between CO_2_ content and winter precipitation. (The Spearman rank correlation coefficients were 0.32, 0.36, 0.37, 0.51, and 0.40 for November, December, January, February, and March, respectively, with a 95% confidence interval for the 120-year period from 1889 to 2008). In this case, however, the correlation was positive, which is evidence for the association of the CO_2_ content and spring soil moisture.

**Figure 10:**
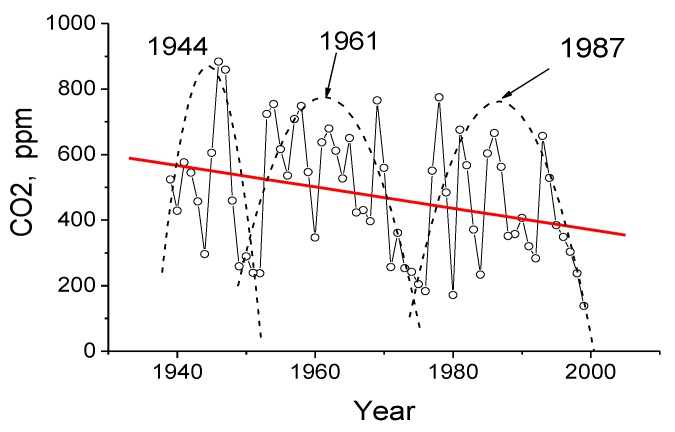
Annual variations in the CO_2_ concentrations (ppm) in the larch disc tree rings (settlement of Chernorud, Lake Baikal, Russia).

For comparison purposes, we have presented the vacuum-desorbed CO_2_ distributions in the disc tree rings of the 300 year old larch and those relating to the discs brought from other regions, specifically from the region located near Lake Baikal. Figure 10 shows annual variations in the CO_2_ content measured in the 61 larch disc tree rings brought from the Baikal region. Using second-order polynomial curves, the tree ring CO_2_ distributions can be grouped together into three time spans. This is indicative of the years wherein the CO_2_ content in these distributions was at its maximum: 1944, 1961, and 1987. Thus, as Figure 10 suggests, the CO_2_ content is characterized by (1) distinct short-term cycles modulated by long-term ones and by (2) a negative trend.

## 4. Conclusions

The results obtained from investigations into the vacuum-desorbed CO_2_ and H_2_O content in larch tree disc rings have shown that considerable portions of these substances are stored in the annual ring wood, *i.e.*, in the tree stems. As the stem CO_2_ originates from respiring cells in the tree stems and roots [6], CO_2_ rise likely to be due to an increase in cell respiration is observed. Our measurements have revealed that (1) the process has a cyclic pattern, with the main cycle being a 4-year period modulated by long-term cycles; (2) 4-year cycles of the CO_2_ distribution in the disc tree ring wood of the 300 year old larch accounts for the 4-year pressure change cycles in gas samples vacuum-desorbed from the disc tree ring wood [14]. The 3.9–4.4-year cycle was observed in dendrochronological series almost without exception. The foregoing cycle was found in different natural processes: in variations in solar activity, atmospheric circulation, precipitation, and temperature [15].

It follows from our findings that the variations in the annual tree ring CO_2_ are accompanied by those observed in the carbon isotope composition. In this case, the trends seen in the carbon isotope composition of the air and larch ring CO_2_ are the same and exhibit a significant correlation. A comparison of the annual CO_2_ variations observed in two larch discs shows that an unfavorable habitat (e.g., very dry climate) changes the sign of the annual CO_2_ trends in the tree rings. Thus, it may be concluded that environmental changes can influence the stem-respired CO_2_ through changes in the cyclicity. Moreover, it can be assumed that the atmospheric CO_2_ rise and the elevated temperatures observed since 1960 have changed the annual diffusion pattern of the stem CO_2_ and caused it to accumulate.

We believe that online *in situ* monitoring of CO_2_ and H_2_O in tree ring cores can provide a wealth of information about a forest system adaptation for climate change and furnish original data about forest health in ecological risk areas. We would like to call attention to the fact that investigations into the gas samples vacuum-desorbed from the disc tree rings carry valuable information about the behavior of gas components in stems.
